# Editorial: Minimally Invasive Dentistry for Caries Management

**DOI:** 10.3389/froh.2022.940177

**Published:** 2022-06-07

**Authors:** Sherry Shiqian Gao, Minquan Du, Diah Ayu Maharani

**Affiliations:** ^1^Department of Stomatology, School of Medicine, Xiamen University, Xiamen, China; ^2^Department of Preventive Dentistry, School and Hospital of Stomatology, Wuhan University, Wuhan, China; ^3^Department of Preventive and Public Health Dentistry, Faculty of Dentistry, University of Indonesia, Depok, Indonesia

**Keywords:** minimally invasive dentistry, dental caries, silver diamine fluoride (SDF), atraumatic restorative treatment (ART), mouth rinse

Dental caries is one of the most prevalent oral diseases in the world. It remains a substantial global burden on health; around 2.3 billion people have untreated dental caries in their permanent teeth [1]. The conventional restorative treatments of dental caries always need the use of the highspeed handpiece and air-water syringe, which can generate aerosol containing bacteria and viruses. Recently, a new coronavirus SARS-CoV-2 has caused the COVID-19 pandemic worldwide. Studies have reported that COVID-19 might spread through aerosols [2]. Minimally invasive dentistry (MID) is a concept defined as the maximal preservation of healthy dental tissues. MID in caries management can embrace all available techniques, including caries diagnosis, risk assessment, caries prevention, and minimally invasive treatments, such as fluoride therapies and atraumatic restorative treatment. Under the COVID-19 pandemic, the concept of MID is particularly valuable because the minimally invasive strategies are always non- or low-aerosol generating procedures, which can lower the risk of virus transmission.

This Research Topic finally has three high-quality articles published. All studies are original research, and they were conducted by wellknown researchers in Australia, Switzerland, and the United States (US). Arrow et al. investigated parents'/carers' perspectives on atraumatic restorative treatment (ART), Hall technique (HT), and dental general anesthesia (GA) using qualitative research design. Focus group discussions were conducted for data collection. This study demonstrated that ART/HT enabled the establishment of a relationship between the clinical team and the child and parents/carers. Parents/carers were equally satisfied with different treatments. But they expressed dissatisfaction more often with GA regarding the issues of timely care, cost of care and accessibility of care. The findings supported that MID approaches are acceptable alternative options to the GA and should be considered for early childhood caries management. Steiger et al. performed a laboratory study using isothermal microcalorimetry (IMC) to investigate the efficacy of mouth rinse containing tin and polyethyleneglycol (PEG-3) tallow aminopropylamine in different concentrations on *S. mutans* biofilms. This study revealed that IMC is a reliable tool to screen the efficacy of antimicrobial agents and biofilms. In addition, increased concentration of tin and PEG-3 will contribute to a better antibacterial outcome. However, higher concentrations may lead to resistance development over time. Therefore, the authors suggested that the choice of mouth rinse should be carefully considered based on whether short or long term use is planned. Ruff et al. conducted an observational pilot study to develop a predictive model of treatment non-response of silver diamine fluoride (SDF) using machine learning. 16S rRNA genes from saliva and plaque samples were amplified and sequenced. The association between operational taxonomic units and treatment non-response was assessed using machine learning algorithms: lasso regression and artificial neural networks. Bivariate group comparisons of bacterial abundance indicate several genera were significantly different between those who responded to SDF therapy and non-responders. The authors make a conservative conclusion that the acid-tolerant and acidogenic nature of oral bacterial species may overcome the antimicrobial effects of SDF; further research based on larger samples is needed.

This Research Topic makes a considerable contribution to the literature on MID research. First, it collected studies with high-quality from researchers in America, Asia-pacific, and Europe. By reading this Research Topic, dental professionals can have an idea of the trend of MID research updates all over the world. Second, the studies in this Research Topic have different study designs, including one laboratory study and two clinical studies from both quantitative and qualitative aspects. This reflects that research on MID for caries management can be multi-dimensional. Laboratory studies are needed to investigate the mechanism of MID approaches for caries management, such as anti-bacterial and remineralising properties. Clinical studies with a quantitative research design are crucial for defining the effectiveness of MID therapies for preventing or treating dental caries. In addition, clinical studies with a qualitative research design can further investigate patients' perspectives regarding MID approaches for caries management. Different study designs help the readers to have a complete understanding of MID strategies used in treating dental caries. Last but not least, this Research Topic introduced several MID approaches for caries management, including mouth rinse, ART and SDF therapy. Using a mouth rinse with anti-bacterial ingredients is effective in preventing dental caries. ART is commonly used for treating caries in primary teeth in a less-invasive way. With fluoride release, ART is also effective in preventing further caries. SDF is a solution containing fluoride and silver ions. Fluoride is effective in enhancing the remineralisation of dental hard tissue, whereas silver has anti-bacterial properties [3]. Therefore, SDF is effective in arresting dental caries. Moreover, the application of the SDF solution is simple and non-invasive; it can be a promising MID strategy for caries management.

In conclusion, this Research Topic identified good studies investigating MID approaches for caries management. Dental professionals and clinicians can refer to those studies when conducting their clinical works.

## Author Contributions

SSG contributed to the initial draft preparation. MD and DM contributed to the review and editing. All authors have read and agreed to the published version of the manuscript.

## Conflict of Interest

The authors declare that the research was conducted in the absence of any commercial or financial relationships that could be construed as a potential conflict of interest.

## Publisher's Note

All claims expressed in this article are solely those of the authors and do not necessarily represent those of their affiliated organizations, or those of the publisher, the editors and the reviewers. Any product that may be evaluated in this article, or claim that may be made by its manufacturer, is not guaranteed or endorsed by the publisher.
